# Potential of small-molecule targeted drugs in combination with CAR-T cell therapy for hematologic lymphomas

**DOI:** 10.3389/fimmu.2026.1792910

**Published:** 2026-04-01

**Authors:** Wenhao Tang, Siyao Yu

**Affiliations:** 1 Department of Pediatrics, West China Second University Hospital, Sichuan University, Chengdu, China; 2 Key Laboratory of Birth Defects and Related Diseases of Women and Children (Sichuan University), Ministry of Education, Chengdu, China

**Keywords:** CAR-T cell, combination therapy, lymphoma, resistance mechanism, small-molecule drugs

## Abstract

As the traditional first-line standard treatment of B-cell non-Hodgkin’s lymphoma, the R-CHOP chemotherapy regimen faces the problem of about 30%-40% of patients progressing into relapsed or refractory disease. Small molecule targeted drugs and CAR-T cell therapy, represented by BTK inhibitors and Bcl-2 inhibitors have achieved breakthrough results in the treatment of lymphoma, but they still face restrictions such as limited single-drug efficacy, drug-resistant recurrence, and toxic reactions. In order to overcome the shortcomings of single therapy, combined treatment strategies have become a research hotspot. This review systematically summarizes the efficacy evidence of the current preclinical and early clinical combined treatment of small molecule targeted drugs and CAR-T cell therapy. The potential synergistic mechanism of the joint application of small molecule targeted drugs and CAR-T cell therapy is discussed, including improving the tumor microenvironment, enhancing the function of CAR-T cells, improving the sensitivity of tumor cells to CAR-T, inhibiting exhaustion, and reducing toxicity. This joint strategy is expected to improve the therapeutic effect and overcome drug resistance. It is a very promising development direction for the treatment of relapsed or refractory lymphoma in the future. At the same time, further in-depth research is needed to promote its clinical transformation and application.

## Introduction

Chimeric antigen receptor T cell (CAR-T) therapy is a major progress in tumor immunotherapy. Genetically modified T cells to specifically identify and remove tumor cells, which has shown remarkable therapeutic effect in a variety of hematologic malignancies (1). The U.S. Food and Drug Administration (FDA) has approved CAR-T products targeting CD19 and B-cell maturation antigen (BCMA) for the treatment of relapsed or refractory (R/R) diffuse large B-cell lymphoma (DLBCL), B-cell acute lymphoblastic leukemia (B-ALL), and multiple myeloma (MM) (2). CAR-T cell therapy can achieve durable remission in some patients, but its overall efficacy still faces challenges. The complete remission rate (CRR) of DLBCL is 43%, while the CRR of follicular lymphoma (FL) can reach 71% (3). Studies show that about 27%–47% of R/R DLBCL patients show primary drug resistance to CD19-targeted CAR-T cell therapy (4, 5), and 30%–50% of initial remission patients eventually have disease recurrence (6). These limitations are mainly due to the lack of persistence of CAR-T cells *in vivo*, immunosuppression in the tumor microenvironment (TME), loss of target antigens (7–9), and treatment-related toxicities, such as cytokine release syndrome (CRS), immune effector cell-associated neurotoxicity syndrome (ICANS), etc. (10).

Although the R-CHOP regimen based on rituximab is the standard first-line treatment for B-cell non-Hodgkin lymphoma (B-NHL) and improves the prognosis of patients, about 30%-40% of patients still develop R/R disease (11). Small molecule targeted therapies such as Bruton’s tyrosine kinase (BTK) inhibitors, B cell lymphoma-2 (Bcl-2) inhibitors and histone deacetylase (HDAC) inhibitors have shown efficacy for R/R B-cell lymphoma. However, the overall prognosis is still poor (12). In order to solve problems of drug resistance and relapse of single treatment, combined therapy is becoming a key strategy to improve CAR-T cell therapy. Studies show that small molecule inhibitors can improve the amplification and persistence of CAR-T cells, improve the sensitivity of tumor cells to CAR-T, inhibit depletion, and reduce toxicity. Preclinical and early clinical studies show that this combination strategy can enhance anti-tumor effect through multiple mechanisms, providing a promising method to overcome CAR-T resistance (13, 14). This paper systematically reviews the application and limitations of CAR-T cell therapy and small molecule drug monotherapy in the treatment of lymphoma, and the research progress of combined treatment of lymphoma. The possible synergistic mechanisms and clinical efficacy are discussed, and the treatment is optimized for patients with R/R lymphoma and research direction is provided.

## Overview of small-molecule drugs for lymphoma

### Bruton’s tyrosine kinase inhibitor

BTK is a key molecule in the B-cell receptor (BCR) signaling pathway and plays a crucial role in the development, maturation and survival of B cells (15, 16). Due to the dependence of B lymphocytes on BTK and their overexpression in various B-cell malignant tumors, inhibiting BTK is also an important way to treat B cell malignant tumors, and BTK has become a promising treatment target. According to the current treatment guidelines, BTK inhibitors are used to treat multiple B-cell lymphomas, including chronic lymphocytic leukemia/small lymphocytic lymphoma (CLL/SLL) and mantle cell lymphoma (MCL) (17). Ibrutinib is the first covalent BTK inhibitor approved by the U.S. FDA for the treatment of R/R MCL. It was approved based on a phase II trial with excellent efficacy. The results showed that the overall response rate (ORR) was 67%, the median progression-free survival (PFS) was 13 months, and the median overall survival (OS) was 22.5 months (18, 19). The success of ibrutinib has also promoted the research and development of a new generation of BTK inhibitors, which aims to improve target selectivity to reduce off-target toxicity and overcome acquired drug resistance. The second generation of covalent BTK inhibitors (such as acalabrutinib and zanubrutinib) exhibit higher selectivity and the same efficacy. Acalabrutinib (approved in 2017) showed 81% of ORR and 48% of CRR in R/R MCL, with a median PFS of 22 months (20, 21). Zanubrutinib (approved in 2019) has an ORR of 84% for the same indication (22, 23). Orelabrutinib was approved for the first time in China in 2020 for the treatment of patients with MCL or CLL/SLL who had received at least one therapy in the past (24). At present, its indications have been expanded to include first-line treatment of newly diagnosed CLL/SLL.

Although BTK inhibitors have shown significant efficacy in B-cell malignancies, drug resistance and adverse reactions are still important clinical problems. Approximately 60% of patients receiving long-term ibrutinib will develop drug resistance, and the prognosis is generally poor (25–27). The off-target effect of BTK inhibitor will cause adverse reactions and limit their clinical application. These reactions mainly include atrial fibrillation (5%-12%), hypertension (5%-13%), and bleeding events (up to 51% in patients treated with ibrutinib plus rituximab) (28, 29). These toxic reactions often require dose adjustments or discontinuation of treatment. Among CLL patients treated with ibrutinib, about 22.8% of patients needed to reduce the dose due to adverse events (AEs), while 20.6% of patients needed to stop taking the drug (30). In another study, after the median duration of treatment was 20 months, 43.5% of patients temporarily stopped treatment due to toxicity, and 17.7% of patients finally stopped treatment permanently (31).

### BCL-2 inhibitor

The BCL-2 protein family is the core regulatory factor of apoptosis, of which BCL-2 is the key anti-apoptotic protein. It is overexpressed in various cancers, promoting tumor survival and progression (32). Abnormal overexpression of survival-promoting BCL-2 family member or abnormal reduction of apoptotic BCL-2 family protein leads to inhibition of cell apoptosis, which is especially common in hematologic malignancies (33). Therefore, BCL-2 family members and their regulatory factors are important target for development anti-cancer drug (34). Venetoclax (ABT-199/GDC-0199) is the first highly selective oral BCL-2 inhibitor, which has been approved by the U.S. FDA for the treatment of CLL and acute myeloid leukemia (AML) (35, 36). In addition to CLL and AML, venetoclax is also used for other hematologic malignancies. In a phase I clinical trial study of R/R B-NHL, the efficacy of venetoclax in different NHL subtypes was shown. MCL reaching the highest ORR (75%), FL is 38% and DLBCL is 18%. The median PFS was 14 months, 11 months, and 1 month, respectively, and there was no significant correlation between the therapeutic response and the BCL-2 expression levels (37). In the multi-center Phase II trial for high-risk R/R CLL with 17p deletion, venetoclax showed breakthrough efficacy, with ORR reaching 79.4% (38). The safety analysis revealed that almost all patients (97%) had AEs during treatment, and most of them were Grade 1-2. 56% of patients have Grade 3–4 AEs, mainly hematologic toxicity. The most common adverse reactions include gastrointestinal reactions and neutropenia (37, 39).

Although venetoclax has shown clinical efficacy in multiple hematologic malignancies, long-term monotherapy can easily induce drug resistance or loss of dependence on target proteins. Intrinsic or acquired drug resistance is the main obstacle to limiting its therapeutic efficacy, and its complex mechanism has not yet been fully elucidated (39, 40). Research demonstrated that up to 50% of patients with TP53 abnormal CLL will have a recurrence of the disease after 2 years after treatment (41). Drug resistance analysis is based on the collection of samples from patients during venetoclax treatment and after recurrence. In DLBCL and FL, long-term drug exposure will activate the AKT pathway, thus upregulating the survival proteins myeloid cell leukemia-1(MCL-1) and BCL-xL, inhibiting the apoptosis-promoting protein Bim, and ultimately preventing apoptosis (42, 43). In addition, lowering the expression of microRNA-377 by increasing the level of BCL-xL will promote the drug resistance of DLBCL and CLL cells (44). The more direct drug resistance mechanism may also be the mutation of the BCL-2 protein itself (e.g., Gly101Val and Asp103Tyr), which directly reduce the binding force of the drug to the target site (45, 46).

### PI3K inhibitor

Phosphatidylinositol 3-kinase (PI3K) is a key lipid kinase involved in regulating cell growth, proliferation, survival, and metabolism. The abnormal activation of PI3K is closely related to the occurrence of a variety of malignant tumors (47). In B-cell malignancies, the PI3K signaling pathway is usually overactivated (48, 49). Several PI3K inhibitors have been developed and approved for the treatment of relapsed or refractory CLL, SLL, and indolent non-Hodgkin lymphoma (iNHL), including FL and marginal zone lymphoma (MZL) (50, 51). Idelalisib combined with rituximab can significantly prolong PFS (20.3 vs. 6.5 months) and OS (40.6 vs. 34.6 months) in patients with R/R CLL (52). Copanlisib in the phase II trial for R/R iNHL, the ORR of iNHL patients reached 59%. The median duration of response (DOR) is 22.6 months. In 2017, the FDA accelerated the approval of copanlisib for the treatment of relapsed FL who have received at least two previous therapies (53). Umbralisib monotherapy treated R/R iNHL patients. The results showed that ORR reached 42.2% in FL, MZL reach 38.9%, and SLL reached 67.9%, with the median DOR is 10 months, and the median PFS is 9.5 months (54).

Although PI3K inhibitors have significant efficacy, safety problems have been observed. Research shows that inhibiting different subtypes may lead to characteristic adverse reactions. For instance, inhibiting PI3Kδ frequently induces autoimmune toxicity, such as colitis, pneumonia, and rash. And inhibiting PI3Kα is usually manifested as hyperglycemia and hypertension (55). These AEs may also lead to the termination or interruption of multiple clinical trials. For example, Phase II studies of idealisib, copanlisib, and duvelisib in patients with R/R iNHL showed that 20%–31% of patients stopped treatment due to AEs, and 19%–34% needed to reduce the dose, 47%–68% experienced a delay or interruptions of medication (53, 56, 57). In addition to safety issues, drug resistance is also a major barrier to the long-term clinical application of PI3K inhibitors. Research shows that tumor cells can escape PI3K inhibition through multiple mechanisms. Tumor cells can bypass the inhibitory effect of PI3K by reactivation of upstream receptor tyrosine kinases (RTKs), mutation or downstream signaling nodes (e.g., AKT or mTOR), loss of PTEN function or compensatory activation of bypass signaling pathways (e.g., the MAPK pathway) (58). There are also researches confirming that feedback signal loop plays a key role in drug resistance mechanisms. PI3K inhibition may release negative feedback regulation on RTKs, leading to the upregulation and reactivation of the downstream pathways, thus reducing the efficacy of monotherapy (50).

### EZH2 inhibitor

EZH2 (Enhancer of zeste homolog 2) is a key epigenetic regulatory factor that controls the normal development and differentiation of germinal center (GC) B cells in the growth center (59, 60). EZH2 is thought to be associated with a variety of cancer types, mainly because its mutation, amplification and/or overexpression are closely related to cancer progression and poor prognosis. In B-cell malignancies, overexpression of EZH2 is more common in aggressive B-cell lymphomas, with an incidence of more than 80% in GCB-DLBCL and FL (61). In view of the core driving role of EZH2 in the development of lymphoma, targeting EZH2 has become a very promising therapeutic strategy (62, 63). Tazemetostat is the first oral EZH2 inhibitor approved by the FDA. Phase I clinical trial data demonstrated that R/R B-NHL patients have achieved 38% ORR (62), of which the CR rate of R/R DLBCL patients is 33% (64). Subsequent phase II studies further confirmed that the efficacy of the drug in FL patients was related to the state of EZH2 mutation. The ORR of mutant patients was 69%, the median PFS was 13.8 months, while the wild-type patients were 35% and 11.1 months, respectively (65). Another phase II study of R/R DLBCL and FL patients also proved that the ORR was significantly higher in mutant-positive patients (66). Both studies have shown that the drug is well tolerated. Common AEs include nausea, fatigue, diarrhea, and hematologic toxicity, with a low incidence of grade 3/4 events (65, 66).

However, the emergence of drug resistance is becoming a major challenge in the clinical application of EZH2 inhibitors. The drug resistance mechanism of EZH2 inhibitors is complex and diverse. The secondary mutation of EZH2 protein (e.g., Y641 and A677G) directly reduces the binding affinity of the inhibitor to the catalytic domain, thus inducing drug resistance (67). Compensation reprogramming at the epigenetic level is very important. SWI/SNF chromatin remodels the complex subunit to undergo functional deletion mutation or expression upregulation, the silent function of PRC2, reactivate the expression of key genes, and drive the occurrence of drug resistance (68). In addition, the continuous activation of the BCR signaling pathway transmits survival signals through the downstream PI3K/AKT and MAPK/ERK pathways, avoiding the inhibitory effect of EZH2, thus maintaining the proliferation and survival of tumor cells (69).

### SYK inhibitor

Spleen tyrosine kinase (SYK) is a key kinase in the BCR signaling pathway and is primarily expressed in hematopoietic cells. It regulates immune signaling by binding to immune receptors (such as the BCR, Fc receptors (FcRs), and C-type lectin receptors (CLRs) or by connecting downstream adaptor proteins that carry immunoreceptor tyrosine-based activation motifs (ITAMs). Abnormal sustained activation of this pathway drives the initiation and progression of B-cell lymphomas and leukemias (70). Abnormal SYK activation has been observed in CLL (71). Therefore, it is considered a highly promising therapeutic target among hematologic malignancies (72, 73). Preclinical studies indicate that SYK inhibition effectively blocks BCR signaling, leading to cell cycle arrest, growth suppression, and induction of apoptosis in tumor cells (74). The feasibility of SYK as a therapeutic target has been clinically validated. Phase I/II trial data for fostamatinib, the first oral SYK inhibitor, demonstrated subtype-specific efficacy in patients with R/R B-NHL. ORR were 22% in DLBCL, 10% in FL, and 55% in SLL/CLL. The overall median PFS was 4.2 months (71). A subsequent Phase II randomized controlled trial (NCT01499303) further demonstrated that some patients with R/R DLBCL (all with GC B-cell or intermediate subtype) achieved clinical benefit (remission or stable disease), with two patients maintaining long-term remission exceeding 6 years (75). A Phase II study of the second-generation SYK inhibitor entospletinib revealed its favorable efficacy in R/R CLL, with a 70.1% PFS rate at 24 weeks, a median PFS of 13.8 months, and an ORR of 61.0%. The most common Grade 3/4 AEs included neutropenia (14.5%) and elevated transaminases (13.4%) (76).

Research on the mechanism of drug resistance is being deepened. After long-term use of SYK inhibitors, tumor cells may compensate for the inhibition of BCR signal conduction by activating alternative survival signaling pathways (e.g., MAPK/ERK or JAK/STAT), thus driving acquired drug resistance (74). The clinical results found that some patients had secondary drug resistance after entospletinib treatment, mainly manifested as recurrent or progressive lymph node enlargement, suggesting that tumor cells may reactivate the downstream signaling pathway of BCR through genetic mutations or epigenetic changes (76).

### Lenalidomide

Lenalidomide is an oral immunomodulatory drug (IMiD), a derivative of thalidomide, which shows multifaceted antitumor effects in the treatment of B-cell malignancies (77, 78). The drug activates and promotes T-cell proliferation by binding to the E3 ubiquitin ligase cereblon (CRBN), thus inducing the degradation of T cell transcription factors Ikaros and Aiolos (79). This drug can also inhibit the secretion of pro-inflammatory factors such as TNF-α and IL-6, and promote the production of the anti-inflammatory factors IL-10, thereby exerting an immunomodulatory role. At the same time, it directly inhibits tumor cell proliferation and angiogenesis, and enhances the cytotoxicity of T cells and natural killer cells (80, 81). When used in combination with antibody drugs like rituximab, it can jointly enhance antibody-dependent cytotoxicity (ADCC) (82). Lenalidomide single drug or combined regimen shows its remarkable clinical efficacy for multiple B-cell lymphoma. The ORR of monotherapy of R/R MCL reached 35%-53%,and was approved by the FDA for this indication (83, 84). In indolent lymphoma, the R² regimen (lenalidomide plus rituximab) demonstrates significantly superior efficacy compared to rituximab monotherapy, markedly prolonging PFS in patients with R/R FL and MZL. It has emerged as an effective chemotherapy-free option for treatment-naive FL with high tumor burden (85). In the setting of aggressive malignancies, the combination of lenalidomide with the anti-CD19 monoclonal antibody (L-MIND study) delivered deep and durable responses in patients with R/R DLBCL, achieving an ORR of 57.5% and a median OS of 33.5 months (86). The overall safety profile of lenalidomide is manageable, with well-defined adverse reaction characteristics. The most common AEs are hematologic toxicities (e.g., neutropenia and thrombocytopenia), whereas non-hematologic toxicities primarily include rash, fatigue, diarrhea, and others (84, 87).

## Overview of CAR-T therapy for lymphoma

After decades of development, CAR-T cell therapy has achieved unprecedented success in tumor immunotherapy, particularly for R/R B-cell malignancies (88, 89). The U.S. FDA has approved seven CAR-T cell therapy products, targeting CD19 and BCMA. Indications cover B-ALL, large B-cell lymphoma (LBCL), MM, FL, and MCL. In second-line treatment for hematologic malignancies, most products achieve an ORR in the 80-90% range, with ORR twice that of conventional therapies. Owing to the highly effective remissions achieved with CAR-T cell therapy, the 2025 NCCN guidelines list two CD19 CAR-T cell therapies (Yescarta and Breyanz) as Level I recommendations for R/R DLBCL and Level II recommendations for R/R MCL (6, 90, 91). In the treatment of B-cell lymphoma, CAR-T cell therapy has shown remarkable clinical effects. In the study of R/R DLBCL patient, axicabtagene ciloleucel achieved an ORR of 54%, and the 5-year OS rate is expected to be 43% (92). In contrast, the 2-year OS rate of standard therapy in historical controls was only 20% (93). CAR-T cell therapy has also demonstrated durable efficacy in other B-cell lymphoma subtypes. In FL, the 2-year PFS rates for axicabtagene ciloleucel and tisagenlecleucel were 63% and 57%, respectively (94). In MCL, the 12-month PFS rates for brexucabtagene autoleucel and lisocabtagene maraleucel were 61% and 53%, respectively (95, 96).

## Limitations of CAR-T cell therapy

**Figure 1:**
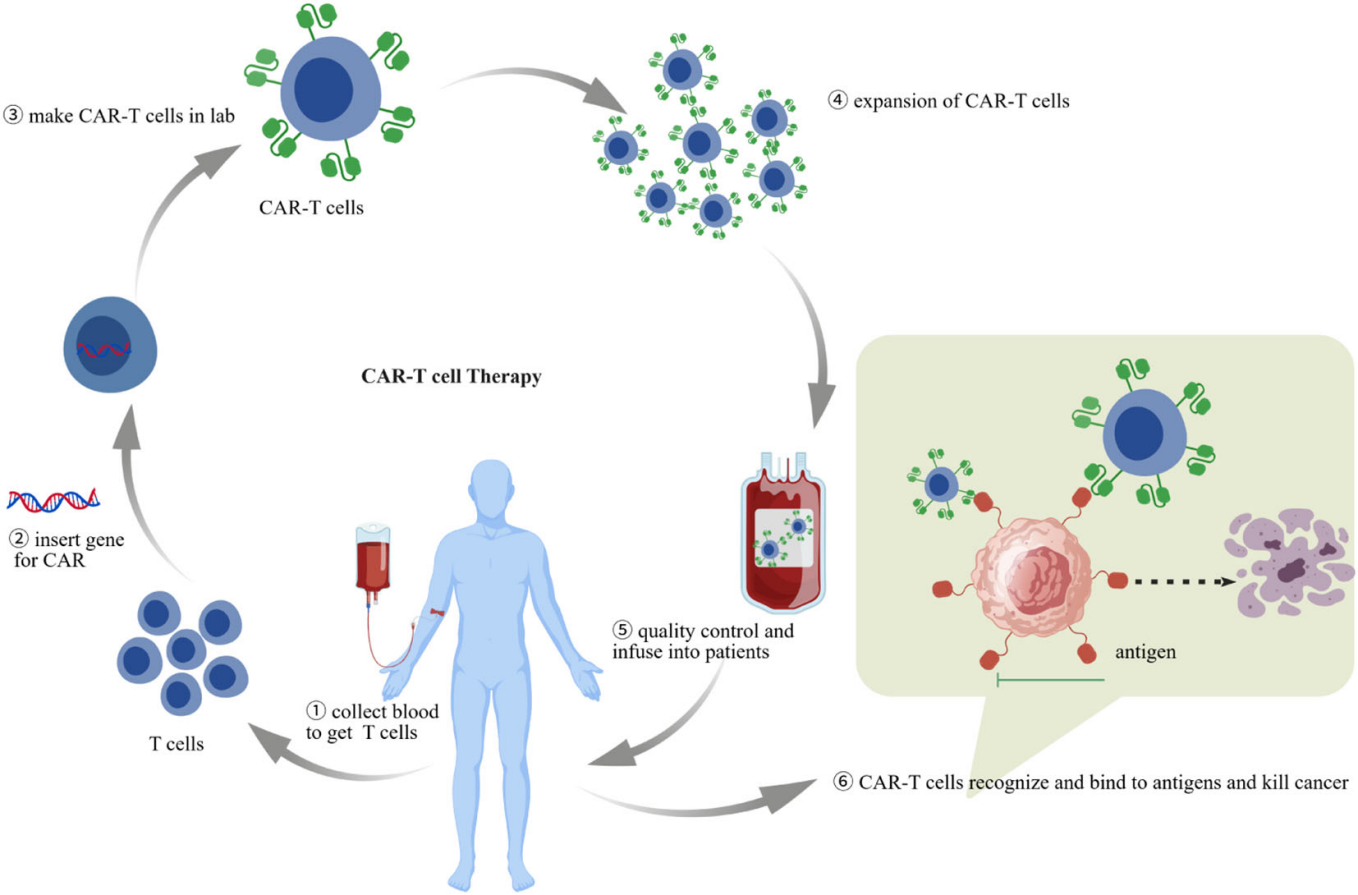
CAR-T cell therapy workflow. including (1) collection of PBMC from patients or healthy donors, (2) T-cell isolation and activation, (3) *in vitro* T-cell gene modification, (4) *in vitro* CAR-T-cell expansion, (5) CAR-T-cell quality control, (6) CAR-T-cell transfusion into patients.

However, CAR-T cell therapy faces numerous challenges, including acute and long-term adverse reactions, limited persistence of efficacy, and drug resistance problems (97). The most significant acute adverse reaction is CRS. Its pathogenesis comes from the large number of activation and proliferation of CAR-T cells, triggering the strong release of pro-inflammatory cytokines, such as interleukin 6 and interferon γ (98, 99). The clinical manifestations of CRS can progress from mild symptoms such as fever and rash to severe reactions such as hypotension, respiratory failure and multiple organ dysfunction. Severe CRS is usually accompanied by an increased risk of CAR-T cell-associated encephalopathy syndrome (CRES) and coagulation disorders (100). In addition to CRS and CRES, we also need to pay close attention to B cell reduction and secondary infection. While eliminating tumors, CAR-T cell therapy may also damage normal B cells, resulting in a decrease in B cell reduction and beta-globulin levels, thus damaging humoral immunity (101, 102). Similarly, in the treatment of T cell malignant tumors, T cell hypoplasia may occur, because the target antigen is usually co-expressed on normal T cells (103). In addition, lymphocyte clearance chemotherapy before infusion will inhibit immune function and increase the risk of infection (104). The long-term adverse reactions of CAR-T cell treatment are mainly manifested as persistent bone marrow and immunosuppression. About 5%-32% of patients may have a serious infection (105). It generally occurs within 30 days after infusion, mainly bacterial infections, especially blood and respiratory infection (106). It may also increase the risk of viral reactivation in patients infected with chronic hepatitis B virus (HBV) (107). At present, all commercial CAR-T cell products need to be prepared using autologous T lymphocytes, which faces multiple challenges. Patients who have received multiple line chemotherapy often have a decrease in the number of T cells and impaired function, which directly affects the feasibility of preparation. The whole process from white blood cell isolation to the completion of the final preparation is complex and time-consuming (usually takes 10–20 days), and the treatment is expensive (108–110). The difference between personalized preparation methods and standardized drug production models makes unified quality control very challenging (111). There are major technical barriers in T cell activation, CAR gene transmission and *in vitro* amplification. The data shows that the preparation failure rate is about 2% to 14% (112, 113)(**Figure 1**).

## Mechanisms of therapy resistance of CAR-T cells

**Figure 2:**
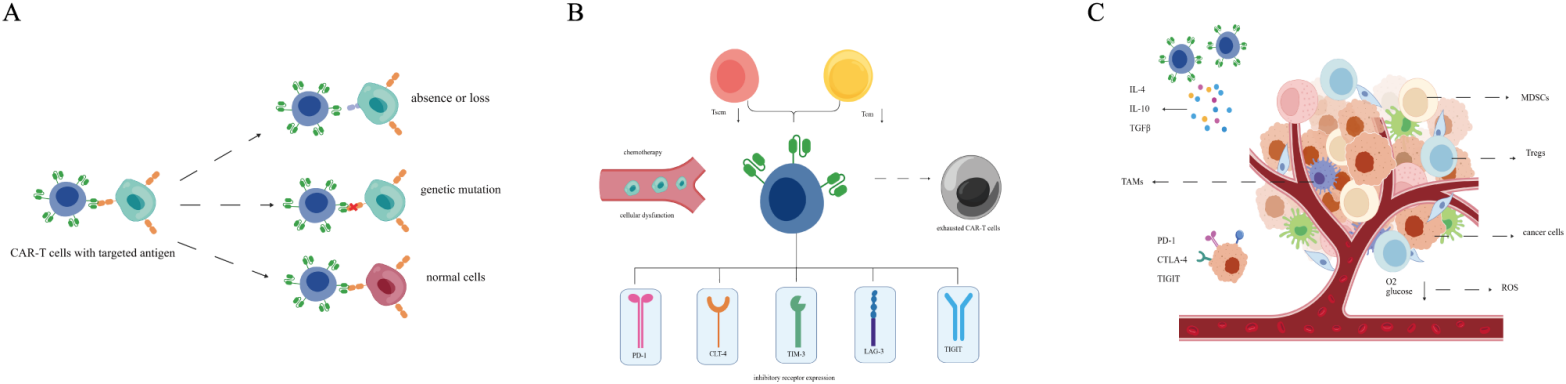
Mechanisms of CAR-T cell resistance and relapse. **(A)** Tumor response: antigen escape, antigen-positive relapse, and gene mutation. **(B)** CAR-T cell dysfunction: drives T cell exhaustion programmes and promotes the increased expression of multiple IRs, impairs the ability to cope with hypoxia and compensatory glucose deficiency, leading to ROS **(C)** the immunosuppressive TME, secrete inhibitory cytokines (IL-10, IL-4, and TGFβ).

Despite significant advances in CAR-T cell therapy for the treatment of hematologic R/R malignancies, high post-treatment recurrence rates remain a major challenge (3). Clinical data show that some patients will have primary drug resistance (no response to initial treatment) or secondary drug resistance (relapse after initial response) (88, 114). The mechanism of drug resistance to CAR-T cell therapy is complex and interrelated, mainly CAR-T cell dysfunction, tumor intrinsic mechanism and the immunosuppressive TME (115, 116) (**Figure 2**).

### CAR-T cell dysfunction

The long-term efficacy of CAR-T cells is closely related to their persistence and functional state in the body, which mainly depends on the degree of differentiation of T cells. Stem cell-like memory T cells (Tscm) and central memory T cells (Tcm) with a low degree of differentiation have strong self-renewal and proliferation capabilities, which contribute to the long-term existence of CAR-T cells and can rapidly agment when the antigen is exposed again. However, highly differentiated terminal differentiation effect cells show limited proliferation ability and increased susceptibility to failure or apoptosis, resulting in a decrease in the persistence of CAR-T cells in the vivo (115). Under continuous antigen stimulation, T cells are easy to enter a state of functional failure (117), manifested as weakened proliferation ability, reduced cytotoxicity, and increased expression of a variety of inhibitory receptors, including PD-1, CTLA-4, TIM-3, LAG-3 and TIGIT (118–120). The expression level of these receptors is positively correlated with the degree of T cell dysfunction (117). The ability of depleted T cells to secrete cytokines (such as IFN-γ, IL-2 and TNF) and cytotoxic function have been significantly reduced. It also expresses transcription factors related to effect T cells and memory T cells, inducing the exhaustion program, inhibiting T cell activation, thus reducing their ability to resist tumor response (121). The quality of the patient’s own T cells is also a critical factor. After multiple cycles of chemotherapy, the quality of T cells is often damaged, the proportion of aging phenotype increases, and the proportion of CD8+ T cells is relatively low (122, 123).

### Tumor antigen escape

Antigen escape is a classic drug resistance mechanism in CAR-T cell therapy, that is, tumor cells escape immune recognition by downregulating or losing target antigen expression. For example, in DLBCL, about 27%-35% of recurrences are related to the loss of CD19 antigen, and similar phenomena have been observed in B-ALL (116, 124, 125). CD19 antigen expression loss can occur through a variety of mechanisms, including gene mutation, gene deletion, selective splicing or epigenetic silencing (125, 126). However, partial recurrence does not involve the loss of target antigens. When the duration of CAR-T cells is relatively short, the tumor cells continue to proliferate under the positive expression of the target antigen, leading to early recurrence. Research shows that this recurrence may be related to the dysfunction of the internal death receptor signaling pathway (e.g., the Fas/FasL pathway) in tumor cells, resulting in their insensitivity to CAR-T cell-mediated killing. In this case, continuous antigen exposure not only cannot effectively eliminate the tumor, but also may accelerate CAR-T cell failure (127).

### Immunosuppression in the TME

TME is a complex ecosystem that promotes the survival of tumor cells, which can reduce the efficacy of CAR-T cell therapy through multiple mechanisms. In hematologic malignancies, the microenvironment contains immunosuppressive cells, including myeloid-derived suppressor cells (MDSCs), tumor-associated macrophages (TAMs), and regulatory T cells (Tregs). By secreting inhibitory cytokines and chemokines, such as TGF-β, IL-10, IL-4 and other factors, the activity and proliferation of CAR-T cells are jointly inhibited (128, 129). Clinical research has further verified the role of this mechanism. In B-NHL, patients with CR have lower levels of TAM, Treg and MDSC in the tumor tissue. Patients who only achieve partial remission (PR) show excessive expression of these immunosuppressive cells and their related chemokine (130). In addition, highly expressed immune checkpoint molecules (such as PD-L1) in TME can bind to PD-1 on the surface of CAR-T cells, directly inducing T cell failure procedures and inhibiting their anti-tumor effect (131). Interferon-γ (IFNγ) can alleviate the immunosuppressive state of TME by regulating immune checkpoints or related cytokines, thus affecting the efficacy of CAR-T cells (132).

## Advances in the combination of small molecule targeted drugs and CAR-T cell therapy for the treatment of lymphoma

**Figure 3:**
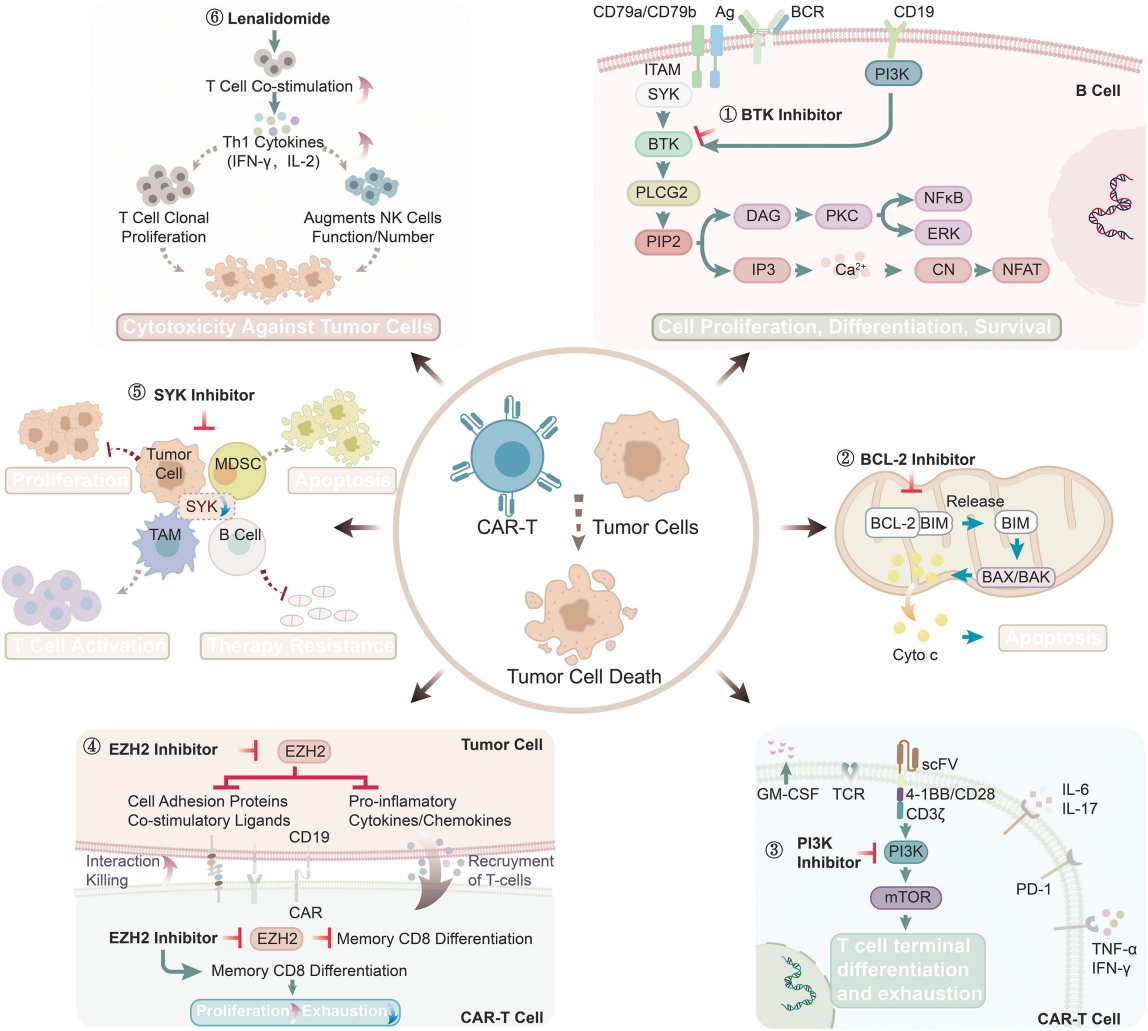
Synergistic mechanism of small molecule targeted drugs combined with CAR-T cell therapy for lymphoma. ①BTK inhibitors block BCR signaling, attenuate CAR-T tonic signaling, delay CAR-T cell exhaustion, reduce M2 macrophage infiltration, promote Th1 polarization, and remodel the tumor microenvironment; ②BCL-2 inhibitors promote tumor cell apoptosis and enhance cytotoxicity of CAR-T cells; ③PI3K inhibitors block Fas signaling-mediated apoptosis, increase the number of CD8+ T cells, enhance anti-tumor cytotoxicity, and prevent cytokine release syndrome; ④EZH2 inhibitors decrease the expression of T-cell inhibitory receptor, increase the frequency of memory CD8+ CAR-T cells, and enhance the activation, expansion, infiltration, proliferation and anti-tumor activity of CAR-T cells; ⑤SYK inhibitors promote T cell proliferation and activation, induce macrophage polarization towards immunostimulatory phenotypes, and reduce immunosuppressive Tregs; ⑥ Lenalidomide enhance the tumor lysis activity of CAR-T cells, increase T cell number, and promote T cells differentiate to CD8+ memory T cells.

CAR-T cell therapy has brought significant clinical benefits to patients with B-cell malignancies, but its efficacy is often limited by CAR-T cell resistance and limited persistence. In order to address this challenge, the scheme of enhancing CAR-T cell treatment through reasonable combination has been widely studied. Although the molecular mechanisms underlying the synergistic effects of small molecule-targeted drugs and CAR-T cell therapy have not been fully elucidated, existing studies have demonstrated the synergistic effects of this combination approach (**Figure 3**). Based on these preliminary mechanistic foundations, investigators have conducted a large number of preclinical studies (**Table 1**), on the basis of which a series of prospective clinical trials and clinical application studies have been promoted to evaluate the safety and feasibility of combination therapies in lymphoma (**Table 2**).

**Table 1:** Preclinical studies of molecular targeted drugs in combination with CAR-T cell therapy.

Types of molecular targeted drugs	Agent	Research focus	Results	Reference
BTK inhibitor	Ibrutinib	Enhance CAR-T cell function and improve tumor clearance in CLL	T cells from three CLL patients who received long-term ibrutinib treatment (≥1 year) exhibited stronger CTL019 expansion *in vitro* and *in vivo*, and this capacity was positively correlated with clinical response. In xenograft models of drug-resistant ALL and CLL, combination therapy significantly improved CAR-T cell engraftment rates, tumor clearance rates, and animal survival rates	(133)
	Ibrutinib	The addition of ibrutinib to anti-CD19 CAR T Cells improves responses against MCL.	CTL019 further enhanced its cytotoxic effect against MCL. Results showed that 80%-100% of mice in the CTL019 plus ibrutinib group maintained long-term remission, whereas only 0%-20% of mice in the CTL019-only group achieved long-term remission (P < 0.05).	(134)
Bcl-2 inhibitor	Venetoclax	Enhance anti-tumor efficacy and survival	In combination with ABT-199 (venetoclax), Bcl-xL overexpressing CAR T cells demonstrate adequate tumor cell clearance and persistence(JeKo-1, Nalm6, and K562 cell lines	(135)
	Venetoclax	Enhance anti-tumor efficacy and survival	Enhances the tumor-killing capacity of CAR-T cells, increasing the killing rate from 47%-63% to 75-88% in multiple lymphoma xenograft models	(136)
	Venetoclax	Reducing activation-induced apoptosis	Increased proliferation of 20BBZ-Bcl-2 CAR-T cells and reduced induction of apoptosis, displaying more potent anti-tumor activity	(137)
	Venetoclax	Improves CAR-T cell quality and anti-tumor efficacy through mitochondrial reprogramming	Increased CD8+ T _SCM_ cell frequency, decreased ΔΨm, reduced T _Regulatory_ (T _REG_) cell frequency, and bolstered effector cytokine production, increasing survival outcome	(138)
PI3K inhibitor	Idelalisib	Optimize the differentiation and function of CAR-T cells in CLL	Enrichment of naïve-like T cells (CD45RA+CCR7+), down-regulation of PD-1 & Tim-3 expression, reduction of TNF-α and IFN-γ release, and restoration of CD4/CD8 ratio to healthy donor levels	(139)
	Duvelisib	Modulates CART cell mitochondrial fusion and epigenetic regulation to enhance antitumor cytotoxicity	Enrichment of T stem cell memory CD8+ CART cell production and alteration of epigenetic pathways for enhanced *in vivo* expansion and anti-tumor activity of CD28 and 41BB CARTs	(140)
	Idelalisib	Increased the sensitivity to CAR T cells in a CD19 low MZL model to enhances CAR-T cell killing activity	PI3K/BTK inhibition in a VL51 cell line model with low CD19 expression levels led to upregulation of CD19 on the VL51 cell surface and did not inhibit tumor cell proliferation compared to the untreated group. CAR-T cells led to 43% growth inhibition on day 4 after co-culture with idelalisib-treated VL51 and completely inhibited growth of ibrutinib-treated VL51	(141)
EZH2 inhibitor	Tazemetostat	Reprogramming the cancer cells at the epigenetic level to enhance immunogenicity	Induce lymphoma immunogenicity, reduce tumor microenvironment immunosuppression, and enhance CAR-T cell function and killing activity; improve CAR-T cell persistence and memory, reduce regulatory T cells, and strengthen antitumor immune responses.	(142)
	Tazemetostat	Reprogramming the cancer cells at the epigenetic level to enhance immunogenicity.	Upregulates genes related to adhesion, B-cell activation, and inflammatory responses, and enhances the activation, expansion, and tumor infiltration of CAR-T cells.	(143)
DNMT inhibitor	Decitabine	Enhance the recognition and killing activity of CD19 CAR-T cells against tumors	Both patients with R/R B-NHL achieved CR, with DFS of 4 months and 2 months, respectively.	(144)
	Decitabine	Enhancing the Antitumor Activity of CART	Compared with conventional CAR-T cells, dCAR-T cells secrete higher levels of IL-2, TNF-α, and IFN-γ and exhibit lower expression of inhibitory receptors, including PD-1, TIM-3, and LAG-3. In contrast, conventional CAR-T cells display a markedly exhausted phenotype.	(145)
HDAC inhibitor		Induction of H3K9 acetylation on the surface of CD20 precursor antigen	Induced CD20 expression on B-Cell malignant cells heightened the cytotoxic activity of Chimeric Antigen Receptor Engineered T Cells	(146)
	Chidamide	Enhance the CART function by modulating the expression of target antigens.	Enhancing the surface expression of CD22 on B-cell tumor cells (including cell lines and primary cells) increased the functional activity of CD22 CAR-T cells *in vitro*. *In vivo* experiments confirmed that upregulating CD22 antigen expression significantly enhances the antitumor effects of CAR-T cells.	(147)
immunomodulatory drug	Lenalidomide	Enhances CAR-T cell function	LEN acts directly on T cells, enhancing antitumor cytotoxicity and boosting interferon-gamma secretion in CAR19 T cells, thereby increasing T-cell activity and infiltration at tumor sites.	(148)
	Lenalidomide	Enhances CAR-T cell function	polarizing CD8+ CAR-T cells to the CD8 early-differentiated stage and Th1 type, reducing CAR-T cell exhaustion and improving cell expansion, modulating the TME.	(149)

**Table 2:** Clinical trials and observations evaluating the combination of CAR-T cell therapy and small-molecule inhibitor.

Types of molecular targeted drugs	Agent	Target	Disease	Phase	Number/publications	Result	Reference
BTK inhibitor	Ibrutinib	CD19	CLL/NHL/ALL	I/II	NCT01865617	Incidence of ≥ grade 3 CRS 0% vs. 26% P = 0.05; Incidence of any grade of CRS 76% vs. 89% P = 0.39; ORR (overall remission rate) 88% vs. 56% P = 0.06; MRD negative rate (IGH sequencing) 83% vs. 60% P = 0.3	(150)
	Ibrutinib	CD19	R/R CLL	II	NCT02640209	Median 42-month follow-up showed a 3-month MRD-negative bone marrow CRR of 44% and 48-month PFS and OS of 70% and 84%	(151)
	Ibrutinib	CD19	R/R MCL	II	NCT04234061	4-month CRR of 80%, a 12-month PFS of 75%, and an OS of 100%	(152)
	Ibrutinib	CD19	MCL	II	NCT06482684	Aims to compare the efficacy of the combination strategy with standard chemotherapy + ibrutinib treatment	(153)
BCL-2 inhibitor	Venetoclax	CD38	DLBCL	NA	Case report	A complete molecular response was obtained, no recurrence of lymphoma	(154)
PI3K inhibitor	Duvelisib	CD19	NHL	I	NCT05044039	Combination therapy was safe and well tolerated, with no grade 3-4 severe CRS and delayed onset of CRS	(155)
EZH2 inhibitor	Tazemetostat	CD19	R/R B-NHL	Ib	NCT05934838	Improved CAR-T cell recognition and killing of lymphoma cells without compromising safety	ongoing
DNMT inhibitor	Decitabine	CD19/CD22	DLBCL	II	NCT03196830	No grade 4 severe CRS; grade 3 CRS occurred in only 7 cases (21.2%), all of which were transient and reversible. Mild ICANS occurred in only 3 cases (9.1%)	(156)
	Decitabine	CD19/CD22	PCNSL	NA	Case report	Obtained a 35-month-long CR without inducing ICANS	(157)
HDAC inhibitor	Chidamide	CD19	R/R B-NHL	NA	Multicenter retrospective study	ORR:44% vs. 20% CRR: 28% vs. 10% median OS: 10.10 months vs. 6.07 months median EFS:6.23 months vs. 1.53 months	(158)
	Chidamide	CD19	R/R B-NHL	I/II	NCT05370547	The primary endpoint was 3-month CRR, secondary endpoints were safety (incidence of AEs), duration of remission, and PFS	ongoing
immunomodulatory drug	Lenalidomide	CD19	DLBCL	NA	Prospective cohort study	C+Len cohort:85.7% achieved a major clinical response, control group:77.8% achieving an objective response (P>0.05)	(159)

### BTK inhibitor

Analysis of preclinical studies in a cohort of CLL patients showed that treatment with ≥5 cycles of ibrutinib resulted in decreased PD-1 expression in T cells and decreased CD200 expression in B-CLL cells, leading to a significant improvement in the *in vitro* expansion capacity of CAR-T cells. In drug-resistant ALL and CLL mouse models, the combination of ibrutinib and CAR-T cell therapy significantly improved the *in vivo* implantation, tumor clearance, and prolonged the survival of CAR-T cells, whereas there was no significant efficacy of either ibrutinib alone or CAR-T alone. This study systematically elucidated for the first time the mechanism by which ibrutinib enhances CAR-T efficacy by improving T-cell function. It lays the mechanism foundation for the clinical application of BTK inhibitor and CAR-T combination therapy (133). A preclinical study on MCL demonstrated that combining CD19 CAR-T (CTL019) with ibrutinib significantly enhances antitumor activity and reduces the risk of relapse compared to monotherapy. The CTL019 + ibrutinib group achieved sustained long-term remission in 80% to 100% of mice, whereas the CTL019 monotherapy group achieved sustained long-term remission in only 0% to 20% of mice (P < 0.05) (134). Several studies have confirmed the efficacy and safety of this combination strategy. A phase I/II study (NCT01865617) initiated by the Fred Hutchinson Cancer Research Center evaluating CD19 CAR-T cells (JCAR014) alone or in combination with ibrutinib in the treatment of R/R B-cell malignancies (CLL, NHL, ALL) and found that ibrutinib in combination with CAR-T reduced the incidence of severe CRS and improved efficacy (ORR 88% vs 56%, P = 0.06) (150). A phase II trial (NCT02640209) in CLL reported that Ibrutinib in combination with humanized CD19 CAR-T (CTL119) was evaluated for the treatment of ibrutinib-treated patients who did not achieve CR. Median 42-month follow-up showed a 3-month MRD-negative bone marrow CRR of 44% and 48-month PFS and OS of 70% and 84%, respectively (151). The results of the phase II TARMAC study for R/R MCL (NCT04234061) showed that ibrutinib in combination with CAR-T cell therapy resulted in a 4-month CRR of 80%, a 12-month PFS of 75%, and an OS of 100%. Deep and durable responses were observed even in high-risk subgroups, such as those with prior BTKi exposure or TP53 mutations, with overall manageable safety (152). Combination therapies are being expanded to patients with first-line, high-risk lymphomas. CARMAN, a randomized controlled, international, multicenter, open-label Phase II trial (NCT06482684) is ongoing. Patients with high-risk MCL received 2 cycles of brief induction therapy with rituximab and ibrutinib followed by 6 months of maintenance ibrutinib to assess the efficacy of short-term ibrutinib induction therapy followed by CAR-T cell therapy compared with standard chemotherapy + ibrutinib therapy (153). In addition, timing of dosing is critical. Initiating BTKi therapy prior to lymphocyte clearance enhances CAR-T cell expansion and function, reduces T-cell depletion and decreases the risk of CRS (160). A study of 24 patients with CLL who failed ibrutinib treatment showed that ibrutinib combined with CAR-T cell therapy showed good results. These patients received ibrutinib pre-treatment and continued therapy for at least 3 months. The 4-week ORR reached 83%, exceeding the 56% ORR in the ibrutinib-naive control group, though the difference was not statistically significant. Although the level of CAR-T cell amplification is comparable, the incidence of CRS in patients treated with ibrutinib is lower than that of patients with CLL who have not been treated with ibrutinib (161).

### BCL-2 inhibitor

In view of the anti-apoptotic effect of Bcl-2 family protein in hematologic malignancies, combining CAR-T cells with inhibitors targeting the pathway has become a key strategy to enhance the therapeutic effect. The current research mainly focuses on two methods. One involves binding exogenous Bcl-2 inhibitors, such as BH3 to simulate venetoclax; the other uses genetic engineering to upregulate the expression of anti-apoptotic protein in CAR-T cells. Among the tested compounds, BH3 mimetics (e.g., venetoclax and ABT-737), IAP inhibitors (e.g., Birinapant), Mcl-1 inhibitors (e.g., S63845), and Smac mimetics have been shown to enhance the sensitivity of tumor cells to CAR-T cells (162). Preclinical research by Harvard Medical School shows that overexpression of the anti-apoptotic protein Bcl-xL in CAR-T cells by genetic engineering significantly enhances CAR-T cell survival *in vivo*, anti-tumor activity, and reduces cellular depletion. In mouse models of lymphoma and leukemia, the modified CAR-T cells not only showed a significant increase in single-agent efficacy, but more critically, a synergistic effect when combined with the BCL-2 inhibitor venetoclax (135). Lee YG et al. demonstrated the same synergistic effect. The killing rate increased from 47%-63% to 75%-88%. This mechanism is mainly achieved by promoting tumor cell apoptosis. Overexpression of BCL-2 protein in CAR-T cells by genetic engineering technology can prolong their survival time and enhance their anti-tumor activity (136). Other studies have further explored the direct enhancement of the anti-apoptosis ability of CAR-T cells themselves through genetic engineering. Compared with traditional 20BBZ CAR-T cells, over-expressing BCL-2 20BBZ-Bcl-2 CAR-T cells show stronger proliferation ability *in vivo* and reduce activation-induced apoptosis and anti-tumor activity (137). Research shows that the synergy of venetoclax on CAR-T cells does not entirely depend on its direct killing of tumor cells, venetoclax regulates the mitochondrial metabolism of T cells by enhancing oxidative phosphorylation and fatty acid oxidation while reducing the mitochondrial membrane potential. This promotes the enrichment of CD8+ Tscm cells and reduces regulatory T cells (138). A Study was the first to investigate the impact of venetoclax on CAR-T efficacy in R/R DLBCL patients. The results showed that venetoclax in combination with CAR-T cell therapy synergistically enhanced the killing of drug-resistant lymphoma cells without affecting CAR-T cell survival. Mechanistically, venetoclax significantly optimized the immunophenotype of CAR-T cells by increasing the ratio of Tn/Tscm and Tcm, while decreasing the frequency of Tregs (163). The timing of the drug of BH3 analogues is very important. Studies show that CAR-T pretreatment before infusion of CAR-T can make tumor cells sensitive by upregulating the expression of CD19 and apoptotic protein. While the drug during or after CAR-T treatment will damage the survival and amplification of CAR-T cells (164). In real clinical, Gong D et al. reported a case of a patient with transformed DLBCL (with p53 mutation) who achieved complete molecular remission and long-term relapse-free survival with CAR-T infusion after receiving bridging therapy with daratumumab and venetoclax, in combination with the GEMOX regimen prior to CAR-T cell therapy (154).

### PI3K inhibitor

The clinically approved PI3Kδ inhibitor idelalisib has shown promising efficacy in the treatment of B-cell malignancies. In addition to inhibiting the BCR pathway, idelalisib modulates T-cell differentiation and function. The results of the study showed that idelalisib can be used to optimize CD19-specific CAR-T cells in patients with CLL, enriching less differentiated naïve-like T cells (CD45RA+CCR7+), with a reduction in the expression of PD-1 and Tim-3, and a restoration of the ratio of CD4/CD8 T cells to the level of a healthy donor. In addition, the *in vivo* efficacy of idelalisib-treated CART cells’ *in vivo* efficacy was demonstrated in a xenograft mouse model (139). It was demonstrated that dual inhibition of PI3K δ/γ isoforms by IPI-145 (duvelisib) during *in vitro* T-cell preparation preferentially expanded CD8+ T-cells (including Tscm and Tcm subpopulations) and enhanced *in vivo* viability and cytotoxicity of CD19-CART, the mechanism may be through MFN1/2-mediated mitochondrial fusion in conjunction with epigenetic reprogramming that promotes PI3K δ/γ inhibitor-mediated T cell expansion *in vitro* (165). Based on these studies, Funk CR et al. adding duvelisib to create Duv-CART cells in the manufacturing process of CAR-T cells also showed the same results. Duv-CART cells expressing CD28 or 41BB co-stimulatory structural domains significantly increased the yield, faster clearance of CLL, and longer persistence of T stem cell memory CD8+ CART cells after transplantation into NOG mice implanted with human CLL cell lines. Significantly improved survival of mice harboring CLL compared to conventionally manufactured CART cells (140). Rojek AE et al. demonstrated that targeting R/R DLBCL that inhibition of the PI3Kγ/δ signaling pathway using duvelisib delayed the terminal differentiation of CAR T cells, increased the proportion of TSCM phenotypic cells, and that duvelisib-induced CAR T cells survived longer, which significantly improved the survival rate of lymphoma-bearing mice. The mechanism is that Duvelisib promotes the nuclear translocation of the transcription factor FOXO1 by inhibiting PI3Kδ, which enhances the expression of memory-related genes (e.g., CCR7, TCF7) (166). In MZL, the use of PI3K or BTK inhibitors can induce CD19 expression upreonization on the surface of tumor cells and increase the sensitivity of CD19 hypoexpression cells to CAR-T cell treatment (141). Some researchers also focus on the study of downstream PI3K effecton Akt to enhance the efficacy of CAR T cells. The FDA has approved capivasertib (pan-AKT inhibitor) for breast cancer (167). Results of preclinical studies for the treatment of hematologic malignancies suggest that ex vivo capivasertib treatment during the T-cell stimulation phase of the manufacturing process enhances the antitumor activity of CAR T cells in a mouse model of B-cell lymphoma (168). A phase I trial (NCT05044039) is currently evaluating the feasibility of duvelisib as a CRS prevention regimen following CAR-T cell therapy. Preliminary data show that duvelisib combination therapy was safe and well tolerated, with no grade 3–4 severe CRS detected, and also effectively delayed the onset of CRS (155).

### EZH2 inhibitor

EZH2 is an enzyme that regulates gene expression, its hyperactivity is common in lymphomas and is associated with tumor development and immune evasion (59). Previous studies have shown that EZH2 inhibition can reprogram tumor cells, enhancing their immunogenicity by upregulating antigen presentation, T-cell co-stimulation, and chemotactic pathways (169). Inhibiting EZH2 can induce the expression of co-stimulatory molecules, such as OX40L and CD80, and chemokines, such as CXCL9 and CXCL10, which have been shown to promote CAR-T cell activation and migration to tumor sites (170). Isshiki Y et al. found that EZH2 inhibitors can reactivate immune synapse function between B-cell lymphoma and T cells, rendering lymphoma sensitive to T-cell immunotherapy. This treatment can reduce Tregs, promote the differentiation of CD8^+^ CAR-T cells toward a memory phenotype, and relieve their exhaustion, ultimately effectively reducing tumor burden (142). Porazzi et al. demonstrated that combining tazemetostat with CAR-T cells resulted in the survival of all treated mice within 40 days. In contrast, the CAR-T-only group exhibited complete mortality within 11 days. The mechanism of action does not involve killing the tumor directly, but rather reprogramming the cancer cells at the epigenetic level to enhance their immunogenicity. This process upregulates immune-related gene expression in tumor cells, thereby enhancing their sensitivity to CAR-T cells. Concurrently, it promotes CAR-T cell differentiation toward a memory-like phenotype, reduces exhaustion, and improves their infiltration into the tumor microenvironment (143). Based on the above preclinical findings, there is a Phase Ib clinical trial (NCT05934838) underway to evaluate the safety and preliminary efficacy of tazemetostat in combination with CD19 CAR-T cells in the treatment of R/R B-NHL in order to improve the outcome of immunotherapy in patients with B-cell lymphomas.

### Epigenetic drugs

In recent years, researchers have found that genomic epigenetic mechanisms, such as DNA methylation and histone modifications, may impair signaling in normal hematopoietic pathways. Therefore, epigenetic modifications are considered important targets for the treatment of leukemia and other hematologic malignancies (171, 172). DNA methyltransferase inhibitors (DNMTis), such as decitabine, are common epigenetic drugs that regulate gene expression by inhibiting DNA methylation (173). Li et al. demonstrated in CAR-T cell therapy that co-culturing CD19 CAR-T cells with lymphoma cells pretreated with decitabine increased surface CD19 expression on lymphoma cells. This enhanced the recognition and killing activity of CD19 CAR-T cells against tumors (144). Wang Y et al. have proved that DAC can also act directly on CAR-T cells themselves. Its proliferation, cytokine release and memory phenotypes are enhanced by epigenetic reprogramming, and the exhaustion after antigen exposure is reduced. CAR-T cells treated with DAC showed stronger and more lasting anti-tumor activity both inside and outside the body (145). A Phase II clinical trial (NCT03196830) evaluated the efficacy of a lymphocyte depletion regimen containing decitabine in combination with CD19/CD22 dual-targeted CAR-T therapy for R/R DLBCL. Results demonstrated that this combination regimen is safe and effective, offering a promising treatment option for this patient population (156). A case of decitabine in combination with CD19/CD22 CAR-T and maintenance therapy for refractory primary central nervous system lymphoma was reported in clinical, in which a CR of up to 35 months was achieved after decitabine-initiated tandem CD19/CD22 CAR-T therapy in combination with PD-1 and BTK inhibitors for maintenance therapy, without inducing ICANS (157).The imbalance of histone acetylation can lead to oncogene activation and cancer suppressor gene inactivation, which is related to tumor progression. Histone deacetylase inhibitors (HDACis) affect gene expression by increasing the level of histone acetylation (174). Preclinical studies show that HDAC inhibitors increase the expression of CD20 antigen by inducing H3K9 acetylation on the surface of the CD20 precursor antigen, enhancing the toxicity of CD20-targeted CAR-T cells to tumor cells pretreated with HDACis (146). Chidamide is the first new oral selective HDAC inhibitor developed in China. A recent study shows that chidamide can specifically upregulate the expression of CD22 on the surface of B cell tumor cells *in vitro* and *in vivo*, and promote the efficacy of CD22 CAR T cells. This mechanism may be related to the increase in CD22 expression by inhibiting HDAC to promote histone acetylation, regulate post-transcriptional modifications, and affect CD22 protein transport and redistribution (147). A multicenter retrospective study of NHL patients after CAR-T treatment failure (55 patients) showed that the efficacy of salvage regimens containing chidamide was significantly better than that of regimens without chidamide. The ORR was 44% vs. 20% and the CRR was 28% vs. 10%. In addition, the chidamide-containing regimen significantly prolonged patients’ median OS (10.10 months vs. 6.07 months) and median event-free survival (6.23 months vs. 1.53 months) (158). Further research shows that impaired NOXA function can lead to drug resistance in CAR-T cells. HDACis pharmacologically enhance the expression of NOXA, increasing the sensitivity of cancer cells to CAR T cell-mediated clearance both *in vitro* and *in vivo* (175). Based on the fact that low expression of NOXA protein may be associated with resistance to CAR-T cell therapy, a phase I/II study (NCT05370547) of the efficacy and safety of a chidamide bridging intervention to improve clinical response to CAR-T cell therapy in R/R B-NHL patients is ongoing.

### Lenalidomide

The combination of lenalidomide and CAR-T cell therapy can directly enhance the function of T cells (176), which has been validated in preclinical models and provides a theoretical basis for clinical trials. In preclinical studies of aggressive B-NHL, lenalidomide in combination with CAR19/20 T cells showed synergistic antitumor effects. This combination regimen enhanced interferon-γ secretion, promoted T-cell activation and tumor infiltration, and significantly reduced tumor load in both *in vitro* and *in vivo* models (148). It was effective in enhancing the function of third-generation CD19 CAR-T cells in both cellular and animal models. The mechanism is to polarize CAR-T cells toward a more favorable early differentiation phenotype, reduce cell depletion, promote cell expansion, and improve the tumor microenvironment for CAR-T cell infiltration (149). A prospective cohort study further demonstrated that in patients with R/R DLBCL, CAR-T therapy followed by lenalidomide maintenance (C+Len) resulted in high remission rates, improved OS at 1 year, and showed favorable safety and tolerability profiles. This approach reduces CAR-T cell-induced hyper-immune activation through the immunomodulatory effects of LEN, thereby reducing hematologic toxicity associated with immune-related adverse events, including CRS (159).

## Safety and toxicity

Currently, the safety and toxicity data of combination therapy of small molecule targeted drugs and CAR-T cell therapy are mainly come from preclinical studies and early clinical trials, and the risk profiles of different combination strategies have not been systematically compared. Available evidence suggests that the overall toxicity of combination therapy is controllable, but some drugs may increase hematologic toxicity or risk of infection at specific doses or timings.

The TARMAC study showed that ibrutinib in combination with CAR-T cell therapy for MCL had a grade ≥3 CRS incidence of only 20%, with no grade ≥3 ICANS (152). Studies of pirtobrutinib as a bridging therapy also showed that all CRS and ICANS were grade 1–2 and did not negatively affect the CAR-T cell phenotype (177). In a clinical trial directly comparing BTKi combined with CAR-T cell therapy with CAR-T monotherapy, there were no significant differences in CRS, ICANS, and hematologic toxicity between the two groups, with only a 2.7% incidence of grade ≥3 CRS (178). It is worth noting that dose-effect analysis of BTKi bridging duration suggested that prolonged exposure (≥2 months), while potentially enhancing efficacy, significantly increased the risk of grade ≥2 CRS and hematologic toxicity, suggesting that dosing timing needs to be carefully controlled (179). The combined strategy of BCL-2 inhibitor venetoclax shows dose-dependent toxicity characteristics. In the sensitive lymphoma model, low doses of venetoclax can enhance the killing effect of CAR-T without affecting the vitality of T cells; while in drug-resistant models, higher doses are required to achieve synergistic effects, but high doses of venetoclax can induce CAR-T cell apoptosis (136). The main safety characteristics of PI3K inhibitor combination treatment are reflected in the preventive effect of CRS. Phase I clinical trials showed that there was no dose-limiting toxicity in Duvelisib combined with CAR-T cell therapy. All CRS were grade 1-2, and no grade 3–4 CRS was observed, and it did not affect the amplification and efficacy of CAR-T cell therapy (155). Despite control of CRS, grade ≥3 ICANS occurred in 11-12% of patients, suggesting that the mechanism of neurotoxicity may be partially independent of CRS. Common adverse events were consistent with PI3Ki alone, with a predominance of hematopenia and no significant toxicity stacking, but there is a need to be concerned about the risk of infections, with one death in the study associated with neutropenic sepsis related (180). For epigenetic modulators, a prospective trial of the HDAC inhibitor chidamide in combination with CAR-T cell therapy (NCT05370547) is ongoing, with safety as a secondary endpoint, and results are not yet available. Considering the potential for thrombocytopenia, neutropenia, and cardiotoxicity associated with HDAC inhibitors alone, vigilance is required for hematologic toxicity overlay and risk of infection after combination with CAR-T cell therapy. Combination strategies with DNMT inhibitors are still in the preclinical stage, and their direct toxicity to T cells and *in vivo* safety have yet to be further evaluated. Combination data for lenalidomide were primarily from retrospective analyses and earlier studies. One study showed that salvage therapy with lenalidomide after CAR-T cell treatment failure resulted in a 76% and 65% incidence of grade ≥3 neutropenia and thrombocytopenia, respectively, without a significant increase in the risk of CRS and ICANS (6% for grade ≥3 CRS and 6% for ICANS), and with enhanced CAR-T cell expansion (181). In another study, lenalidomide combined with other agents as a pre-CAR-T bridging treatment for first-line high-risk LBCL showed a 28% CRS rate and all grade 1, with no increased risk of neurotoxicity (182). Several clinical trials (e.g., NCT06414148, NCT06762431, NCT05797948) are currently evaluating the safety of lenalidomide as a consolidation or maintenance therapy after CAR-T cell therapy, with results not yet available. Therefore, future research should focus on conducting more in-depth, large-scale clinical studies to elucidate the toxicity profiles and safety characteristics of combination therapies.

## Discussion

CAR-T cell therapy is an emerging cancer treatment with a broad prospect for hematologic lymphoma. However, its treatment of poor tolerance and disease recurrence is restricted. To overcome these obstacles, the combination of CAR-T cell therapy with other treatments, especially small molecule targeted drugs, has become a research hotspot for improving efficacy and reducing adverse reactions. This study deeply explores the combined application and mechanism of CAR-T cells and small molecule targeted drugs. This combined therapy can enhance the anti-tumor activity of CAR-T cells and improve the treatment response rate and survival rate. It provides new insights and strategies to promote the clinical application of CAR-T cell therapy in hematologic malignancies.

The synergistic mechanisms between the two therapies remain incompletely elucidated, limiting the rational design of combination treatment regimens. The timing, sequence, and dosage of administration also influence efficacy and safety. Pretreatment of tumor cells with small-molecule drugs (e.g., venetoclax or tazemetostat) prior to CAR-T cell infusion has been shown to synergistically upregulate CD19 antigen expression on tumor-cell surfaces or to reprogram cells into a more immunogenic state (143, 164). Song et al. found that the addition of a combination of three PI3K/AKT pathway inhibitors to *in vitro* cultured/modified CAR-T cells significantly elevated the number of Tscm in the CAR-T cell population, and the antitumor effect of Tscm cells lasted longer than that of fully differentiated T cells (183). Results from the duvelisib Phase I study of CRS prevention in patients with NHL treated with CAR-T showed a possible reduction in the incidence of severe CRS. Grade 3–4 CRS was not observed, but 44% of patients developed ICANS (155). With the intensive use of technologies such as CRISPR screening and single-cell sequencing, combination therapies provide a basis for optimizing CAR-T protocols. The new generation of smart CAR-T cells overcomes drug resistance through bispecific CARs and integrates modules such as drug-induced switches and suicide genes for potentiation and safety control. A study has developed lenalidomide-induced safety switches for CAR T cell therapy that are well tolerated, exhibit *in vitro* proliferation, and tumor-killing functions comparable to those of conventional CAR-T cells. It can be rapidly cleared by adding lenalidomide (184). In addition, FasTCAR-T cells obtained by optimizing the preparation process (e.g., by shortening the *in vitro* culture time) have demonstrated superior expansion capacity, persistence, and antitumor activity. A study was conducted to evaluate the feasibility of CD19- and BCMA-dual-targeted CAR-T cells (GC012F) produced via a novel next-day preparation FasTCAR-T process for the treatment of r/r B-NHL. Results showed an ORR of 100% and a CRR of 77.8% at 3 months. Mainly grade 1–2 CRS, one case of grade 3 CRS, no ICANS observed (185).

In conclusion, the combination of molecular targeted drugs and CAR-T cell therapy broadens the treatment options for R/R hematologic lymphomas. Combined treatment is not a random combination, but should follow scientific and reasonable principles, especially in terms of drug resistance and the safety of CAR-T cell treatment. Future research should focus on clarifying the intrinsic mechanism of synergy, determining the best treatment plan, and effectively controlling adverse effects. In this way, patients can benefit safely, effectively and cost-effectively.
